# Supplementary material for: Are carbon emissions trading and green financial instruments synergistic? -Comprehensive quantitative research based on content analysis
Source: PLoS One. 2024 Mar 7;19(3):e0298601. doi: 10.1371/journal.pone.0298601 (PMC10919601; doi:10.1371/journal.pone.0298601)
Supplement: S1 Data — (ZIP) [file pone.0298601.s001.zip › Data/地级市Z_F工作报告.zip!/╔╜╬≈/╠1⁄2╘¡╩╨/└┤╫╘╣1⁄2╓┌║┼í╛┬φ┐╦╩2╛▌═°í┐_2019.docx]

各位代表：

现在，我代表市人民政府向大会报告工作，请予审议，并请政协委员和其他列席人员提出意见。

一、2018年工作回顾

2018年是全面贯彻党的十九大精神的开局之年，是谱写文明开放富裕美丽太原新篇章的起步之年，在市委坚强领导下，全市上下以习近平新时代中国特色社会主义思想为指导，深入贯彻党的十九大精神和习近平总书记视察山西重要讲话精神，认真落实党中央、国务院和省委、省政府以及市委决策部署，全力做好稳增长、促改革、调结构、惠民生、防风险工作，经济社会发展取得新进步。全市地区生产总值增长9.2%，规模以上工业增加值增长10.8%，一般公共预算收入增长19.7%，固定资产投资增长26.2%，社会消费品零售总额增长8.1%，城乡常住居民人均可支配收入分别增长7%和8.1%，城镇新增就业9.5万人，农村劳动力转移就业1.32万人，城镇登记失业率3.33%，居民消费价格涨幅1.8%。空气质量优良天数比例、煤炭产业占比降低率两项省考核指标虽未完成年度目标任务，但也取得较好成绩。其他约束性指标都圆满完成了年度目标。

一年来，我们主要做了以下工作：

转型发展迈出坚实步伐。开展转型项目建设年活动，完成转型项目投资772.61亿元，占全市固定资产投资的63.4%。大力实施工业强市战略，富士康产值迈上800亿元新台阶，太钢双相不锈钢筋在港珠澳大桥批量应用，阳煤化工新材料、比亚迪动力电池、江铃重汽宽体驾驶室整车、太钢碳纤维二期等项目建成投产，清徐精细化工循环产业园开工建设，战略性新兴产业增加值增长16.6%。华润万象城正式开业，青龙古镇对外开放，太山和天龙山基本完成景区提升改造，华夏文明主题公园9个项目完成主体工程。加快发展会展经济、金融服务、现代物流、商业综合体、高端酒店，初步形成现代服务业集聚态势，太原成为全国流通领域现代供应链体系建设试点城市。推进农业供给侧结构性改革，南部城郊农业示范区建设进展顺利，设施蔬菜、葡果种植面积分别增至11.88万亩、25万亩，玉米种植面积调减25万亩。永丰禽业等9家企业在省股权交易中心“农业板”正式挂牌。农村集体产权制度改革试点工作得到中央农办的肯定。

创新引领作用逐步增强。我市成为首批建设国家可持续发展议程创新示范区城市，在哥本哈根首届全球绿色目标伙伴2030峰会等国际平台介绍“太原经验”。成功举办全国首届军民融合发展高峰论坛，太原军民融合创新小镇揭牌。支持科研院所、高校和企业协同创新，智奇铁路、科达自控2家企业被列入国家智能制造试点，国家级企业技术中心达到13个，科技型中小企业、高新技术企业分别增至1926家、966家。树立激励创新转型鲜明导向，设立10亿元科技创新资金和10亿元人才发展资金，重奖太钢 T800 研发团队、太原锅炉集团等，迁入各类人才及家属4万余人，新建院士工作站14个，引进两院院士10名，科技创新实力不断增强，科技支撑作用进一步显现。

城市功能品质持续提升。组织编制生态修复、城市修补专项规划和晋阳湖、西山、汾河等重点片区规划，在全省率先实施“五规合一”。加大城市路网改造力度，“九河”快速化改造全面完成，天龙山路等59项207公里市政道桥工程基本完工，地铁2号线一期实现“洞通”。环卫产业基地餐厨垃圾处理等项目建成投运。青运村、滨河体育中心等9个二青会场馆基本完工。启动7个城中村改造，拆除旧村211万平方米。新开工棚户区安置房2.05万套，基本建成5.27万套。深入开展全国文明城市创建活动，“九乱”整治成效明显，整治背街小巷437条、老旧小区813个、集贸市场55个，城市更加宜居宜业，城市品质进一步彰显。

改革开放活力加快释放。综改示范区32条改革经验向全省复制推广。清徐经济开发区扩区、阳曲现代农业产业示范区获批。完成中央、省驻并国有企业“三供一业”分离移交。完成农村土地承包经营权确权颁证。国有土地二级市场改革试点通过国家验收。出台支持民营企业发展30条，实行市级领导干部联系民营企业工作制度，新增市场主体8.77万户，增长11.97%。组团参加“山西品牌丝路行”“津洽会”“海交会”等活动，建立10个海外工作联络站，与塔吉克斯坦胡占德市正式结为友好城市，与韩国顺天市签署友城意向书。成功举办城市能源低碳与可持续发展论坛、人民网2018大学校长论坛和2018《财富》 CEO 峰会。

三大攻坚战扎实推进。加强重大风险防范化解，坚决打击非法集资，守住不发生区域性金融风险的底线。决战决胜脱贫攻坚，深入开展对口帮扶，投入1.45亿元实施惠民项目516个，娄烦86.88兆瓦和阳曲10兆瓦光伏扶贫电站并网发电，惠及157个贫困村2487个贫困户，阳曲县脱贫成果不断巩固，娄烦县具备脱贫摘帽条件，社会扶贫“一网三超”经验在全省推广。持续改善省城环境质量，市区空气质量综合指数下降9.2%， PM 2.5浓度下降10.6%，建成区基本消除黑臭水体，地表水优良断面率达55.56%，营造林完成43.7万亩，建成区绿化率、绿地率、人均公园绿地面积分别增加1.4个百分点、1.35个百分点和1.88平方米。狠抓中央和省环保督察反馈意见整改，完成配合中央和省环保督察“回头看”，33批822件群众反映问题整改完毕。

在发展中保障和改善民生。推进教育均衡优质发展，五中、成成中学等新校区基本建成，新建扩建幼儿园21所。加快城乡义务教育一体化改革，缩小城乡义务教育差距。在全省率先实施学校内涵提升工程，建设高素质专业化教师队伍。持续实施公办小学免费托管服务，27.9万学生家庭受益。推进医疗基础设施建设，市中心医院、人民医院、妇幼保健院等新院区基本完工。县域综合医改“阳曲样板”在全国推广。提升社会保障水平，康宁医院、儿童福利院、救助站完成主体工程，城乡低保标准、人均补差额度全省最高。加快文化体育事业发展，推出《于成龙》《关公》等一批精品力作，举办“紫禁风华——2018太原·故宫文物展”。成功承办第十五届省运会，太原代表团取得历史最好成绩，圆满完成二青会“带妆彩排”。推进平安省城建设，“扫黑除恶”专项斗争成果显著，在全国省会城市位列先进。坚守安全红线不动摇，各类生产安全亡人事故起数和死亡人数分别下降31.6%和33.6%，社会大局保持稳定。

政府自身建设不断加强。自觉接受人大依法监督、政协民主监督，办理人大代表建议286件、政协提案518件。全面推行企业投资项目承诺制。不动产房产交易流程精简30%。营业执照实现“三十证合一”，企业开办时间压缩至5个工作日。深化拓展“13710”工作制度，行政效能持续提高。深入推进“两学一做”学习教育常态化制度化，坚决贯彻中央八项规定精神，驰而不息纠正“四风”，干部队伍作风进一步转变，干事创业氛围更加浓厚。

各位代表，过去一年取得的成绩，是我们坚持以习近平新时代中国特色社会主义思想为指导，坚决贯彻落实党中央、国务院决策部署的结果，是省委、省政府坚强领导、大力支持的结果，是市委直接领导、正确决策的结果，是全市上下团结一致、奋力拼搏的结果。在此，我代表市人民政府，向全市人民，向各民主党派、工商联和无党派人士，向各位人大代表、政协委员，向驻并部队、公安民警和中央、省驻并单位，向所有关心支持太原改革发展的各界朋友，表示崇高的敬意和衷心的感谢！

在肯定成绩的同时，我们也清醒地认识到，我市发展仍面临一些困难和挑战，主要表现为：新产业新动能支撑作用有待加强，转型创新步伐还需加快；引领性改革亮点不多，开放的步伐还不够大；城市管理精细化水平有待提高，文明城市创建任务艰巨；民生领域短板亟待补齐，人民群众关心的热点、难点问题还需进一步破解；污染防治压力较大，生态环境保护任重道远；营商环境有待进一步优化等。对此，我们要坚持问题导向，采取有力措施，切实加以解决。

各位代表，问题是时代的声音，发展是时代的要求。越有风险挑战,越需要坚定信心；越是任务艰巨,越需要斗志昂扬。我们坚信，只要高举习近平新时代中国特色社会主义思想伟大旗帜，调动一切可以调动的积极因素，凝聚一切可以凝聚的智慧力量，团结一心，众志成城，就一定能够走出一条富有太原特色的率先发展、振兴崛起之路！

二、2019年工作安排

2019年是中华人民共和国成立70周年，是全面建成小康社会关键之年。今年大事多、喜事多，特别是第二届全国青年运动会将在我市召开，做好今年工作意义重大。今年政府工作的总体要求是：以习近平新时代中国特色社会主义思想为指导，全面贯彻党的十九大和十九届二中、三中全会精神，深入贯彻习近平总书记视察山西重要讲话精神，树牢“四个意识”，坚定“四个自信”，坚决做到“两个维护”，认真落实中央经济工作会议、省委十一届七次全会、省委经济工作会议和市委十一届六次全会暨经济工作会议精神，统筹推进“五位一体”总体布局，协调推进“四个全面”战略布局，坚持“一个指引、两手硬”的工作思路，落实省委对太原提出的“两个走在前列”“双提升”目标要求，在市委的坚强领导下，坚持稳中求进工作总基调，坚持新发展理念，坚持推动高质量发展，坚持把供给侧结构性改革与转型综改试验区建设相结合作为经济工作主线，坚持深化市场化改革、扩大高水平开放，加快构建现代产业体系，继续打好三大攻坚战，着力激发微观主体活力，统筹推进稳增长、促改革、调结构、惠民生、防风险工作，进一步稳就业、稳金融、稳外贸、稳外资、稳投资、稳预期，提振市场信心，保持经济持续健康发展和社会大局稳定，不断增强人民群众获得感、幸福感、安全感，在“两转”基础上全面拓展新局面，推动谱写文明开放富裕美丽太原新篇章取得新成效，为全面建成小康社会收官打下决定性基础，以优异成绩庆祝中华人民共和国成立70周年。

主要预期指标是：地区生产总值增长8%左右，规模以上工业增加值增长10%，固定资产投资增长10%，社会消费品零售总额增长7.5%，一般公共预算收入增长8%，城乡常住居民人均可支配收入分别增长6.5%和6.5%以上，居民消费价格涨幅控制在3%左右，城镇新增就业8万人，城镇调查失业率、城镇登记失业率分别控制在6.5%和4.2%以内。不折不扣完成省下达约束性指标任务。

做好今年政府工作，要把握好“六个必须”：

必须坚持在党的领导下推动政府工作新发展。坚持把党对一切工作的领导贯彻到政府工作各方面全过程，全面贯彻落实党中央、国务院和省委、省政府以及市委的各项决策部署，聚焦“示范区”“排头兵”“新高地”三大目标，自觉将太原工作纳入全省大局，找准定位，主动沟通，获取支持，推动转型，全面拓展党的建设和各项事业新局面！

必须坚持在加快经济转型中抢抓新机遇。把“巩固、增强、提升、畅通”八字方针贯穿经济工作始终，正确处理规模与质量、速度与效益、增长与转型、生产与生态等关系，在新一轮科技革命和产业变革中勇立潮头，唱响工业强市主旋律，重塑太原产业竞争新优势！

必须坚持在加快创新发展中增添新动能。创新是引领发展的第一动力。要下好创新“先手棋”，发挥省城创新资源集聚优势，建立以企业为主体、市场为导向、产学研深度融合的技术创新体系，塑造更多依靠创新驱动的引领型发展模式，为高质量发展注入源源不断的动力！

必须坚持在深化改革开放中体现新担当。改革开放是“关键一招”。要以敢为人先的改革意识、兼收并蓄的开放胸襟、攻城拔寨的责任担当，进一步解放思想，以改革促发展，以开放增活力，坚决扛起“补考”的历史责任，坚决担好“赶考”的时代使命！

必须坚持在回应群众关切中展现新作为。百姓笑脸是最美的画面。保障和改善民生是经济社会发展的出发点和落脚点，是人民政府的职责所在。要把人民群众关注的热点作为政府工作的重点，把人民群众的小事当作政府工作的大事，让民生更好地顺应民心，让市民生活一年更比一年好！

必须坚持在提升行政效能中营造新环境。营商环境就是生产力。牢固树立为市场主体和纳税人服务的理念，把市场主体满意度作为政府工作的衡量标尺，在更大范围、更宽领域先行先试一批改革举措，不断提升制度环境软实力，努力打造营商环境新高地！

今年，要重点抓好以下工作：

（一）全力实施创新驱动战略，推动制造业高质量发展。制造业是现代产业体系的支柱，也是推动实体经济高质量发展的关键。要深化转型项目建设年活动，以高端化、智能化、绿色化为方向，以推动集群化发展为路径，以转型综改示范区为主战场主引擎，持续引进和布局一批全局性、基础性、战略性、牵引性强的大项目好项目，全力提升制造业整体素质和竞争力。

打造新兴产业集群。按照龙头带动、链式布局、研发支撑、园区承载思路，推动产业规模化、集群化发展。要推进阳煤太化尼龙66、银邦金属材料等项目建设，确保太钢高端碳纤维千吨级三期项目年底前完成设备调试，镍基合金二期项目年内开工，打造以太钢、阳煤太化等为代表的新材料产业集群。要推进东杰智能装备、京丰电务等项目建设，确保明豪模具一期工程3月正式投产，加快推进二期建设，打造以太重、东杰、明豪等为代表的高端装备制造产业集群。要加快推进比亚迪新能源动力电池及电动客车、江铃重汽新能源重卡、威马新能源汽车等项目建设，积极对接宝能汽车板块和滴滴出行科技公司，推动宝能整车项目和滴滴共享汽车产业园尽快开工建设，打造以比亚迪、江铃重汽、威马等为代表的新能源汽车产业集群，初步形成新能源汽车研发、生产、销售完整产业链。要推进中车太原公司大养机械、城轨车辆造修、铁路重载造修等项目建设，支持晋西、智奇、太重轨道等企业提升车轴、高速轮对、齿轮箱及转向架等关键零部件研发能力，打造以中车、晋西、太重等为代表的轨道交通产业集群。要支持龙芯安全可靠信息产业基地引入华为、神州数码、方正国际等企业关键技术，集聚培育安全可靠信息产业生态。推进中科曙光先进计算中心二期、中电科电子信息产业园等项目建设，打造以富士康、龙芯等为代表的新一代信息技术产业集群。要抓住我省成为国家通用航空产业发展示范省的新机遇，推进飞机拆解基地项目建设，与中国商飞深度合作，积极引进国产大飞机完工中心，打造通用航空产业集群。此外，要积极推进高端柔性显示屏、中化国际新能源电池和膜材料等项目尽快落地建设，加快培育人工智能、大数据、物联网等产业集群，逐步成长为新的优势产业，形成支撑转型的新动能。

改造提升传统产业。落实省委、省政府产业竞争力提升和环保提标的工作要求，通过装备升级换代、工艺流程再造、产品结构优化、绿色循环提升，促进传统产业转型升级。要加快推进太钢棒线材生产线、高端冷轧取向硅钢、中板生产线智能化升级改造等项目建设，提升冶金钢铁行业精深加工水平。要推进全面满足特别排放限值的清徐精细化工循环产业园建设，形成“煤焦气化”高端产业链，实现清洁化、循环化、智能化转型发展。

提升科技创新能力。加快建设国家可持续发展议程创新示范区，推进水、大气环境治理领域标准化体系建设，初步构建“标准化+可持续”的“太原模式”框架。要加强对内对外交流合作，发挥山西煤化所、山西大学、太原理工大学等省城科技资源优势，加强与中科院、同济大学、中关村等省外科研院校、创新基地深度合作，加速产学研成果转化。扶持企业技术中心建设，谋划布局一批重点科技攻关项目，力争在氢能、碳材料等领域取得新突破。要全面落实人才兴市战略，用足用好专项资金，加快推进人才公寓、院士工作站、博士后流动站建设，激发引进一个高端人才、带来一个创新团队、做强一家企业、做大一个产业的链式效应。

加快军民融合发展步伐。习近平总书记把军民融合发展上升为国家战略，为我们加快高质量转型发展提供了新的历史机遇。要认真落实省军民融合发展推进大会安排部署，抓紧申报国家级军民融合创新示范区，打造特色鲜明的军民融合“太原模式”。要加快筹备设立军民融合产业引导基金，支持驻并银行设立军民融合专营支行，建立军民融合专家库，为军民融合技术成果转化和项目实施提供资金人才保障。要加快“一区两园”基础设施建设，推动总投资百亿元兵器集团国家级示范产业园签约落地，争取更多军民融合项目在太原布局建设。

（二）全力提升现代服务业有效供给，稳步扩大消费需求。习近平总书记指出，消费需求是国内最终需求，其规模扩大、质量提升将对投资需求起到引领和倍数作用。要紧紧抓住消费升级这一重要机遇，多渠道扩大服务供给，实现服务业整体提升，内需潜力持续释放。

进一步推动生活性服务业向便民化和高品质转变。要发挥食品街、中正天街、北美 N1 文创街等特色商业街引领作用，打造太原特色消费品牌。加快发展便利店、家政服务等社区商业，提升“十五分钟便民商圈”服务功能，让市民生活更加方便舒心。要发展数字商务，壮大贡天下、乐村淘等本土电商，推进学府园区国家级电商示范园、综保区跨境电商基地、小店区电子商务服务中心等发展。要培育中高端消费增长点，加快推进远大购物、苏宁广场、新城吾悦、宜家家居、奥特莱斯等城市综合体项目建设，满足多层次消费需求。

进一步推动生产性服务业向专业化和价值链高端延伸。要加快推进山西智用区块链中心、山西健康医疗云等数据中心建设，以大数据推动企业设计、生产、管理、服务全过程智能化。要加强消费品工业发展，推进益海嘉里项目建设，培育以锦波生物胶原蛋白为创新核心的生物和日化品产业，塑造消费品工业品牌。要加快发展金融科技、咨询中介、现代物流等高价值服务业，推进中海、信达、京东山西亚洲一号等项目建设，为实体经济提供质量更优、效率更高的服务支持。

进一步推动文化旅游深度融合发展。聚焦国家全域旅游示范区建设，参与全省三大旅游板块开发，整合文旅优质特色资源，推动康养产业发展，把文化旅游产业打造成战略性支柱产业。要加快“大景区”建设，以创建“晋祠——天龙山”国家5A级景区为重点，加快推进太原古县城、千年府衙、青龙古镇、华夏文明主题公园等项目建设。要高标准做好全省旅发大会配套服务，加强旅游公共服务设施建设，提升旅游服务质量和水平，扩大城市知名度。

（三）全力推动改革向纵深发展，持续提升对外开放水平。进一步推动四梁八柱性质的改革走深走实，以改革“一子落”带动转型“满盘活”，以高水平开放为转型发展提供强大动力，以改革铸就自信，以开放迎接未来。

进一步推进重点领域改革。要加快国资国企改革，推进混合所有制改革，做好“三供一业”分离移交后维修改造工作。要深化金融体制改革，加快资本市场发展，增加直接融资规模，完善普惠金融服务体系，推动更多企业上市挂牌。要深化财税体制改革，推进事权与支出责任划分改革，按照不低于5%的比例压减一般性支出，严格财政支出管理，用有限的财力办更多的民生实事。要全面完成机构改革，做好人员转隶、资产处置等工作，确保改革期间有条不紊、扎实推进，改革后激发活力、提高效率。要实质性推动阳曲、清徐撤县设区。

进一步加快开发区改革创新。全面深化“三化三制”改革，做好园区承载、项目集聚、企业引领这篇大文章，加快形成产业配套、资源共享、效率倍增的发展新格局。综改示范区要完成第三轮内部改革，调整优化机构职能设置，提升完善“一网通办”，建设高效政务服务体系，再形成一批可复制可推广的制度成果。加快推进不锈钢园区、清徐经济开发区、阳曲现代农业产业示范区、西山生态文化旅游示范区改革发展。

进一步构建对外开放新格局。深度融入“一带一路”建设，主动参与和承办“山西品牌丝路行”“山西品牌中华行”，变“交通走廊”为“经济走廊”。要落实对外贸易主体培育三年行动计划，支持特色优势外贸企业做大做强。全面实施准入前国民待遇加负面清单管理制度，推动更多外资项目落地。要认真落实出口退税和信保保费补助等政策，开拓东南亚等海外市场。要完善太原武宿综合保税区功能，申建进境水果、冰鲜指定口岸查验场，拓展保税加工、保税物流、检验维修、国际结算等新业务。积极创建国家跨境电商综合试验区。

进一步加大招商引资力度。要着眼招大引强，积极参加“进博会”“中博会”“厦洽会”等招商活动，瞄准世界500强，开展“点对点”精准招商。要持续补链延链，进一步细化完善招商图谱，引进关键环节、核心企业、上下游配套，形成协同发展的“雁阵效应”。要狠抓跟踪服务，落实好市领导对接服务转型项目坐班及协调例会制度，及时研究解决突出问题，加快项目进度。

进一步深化“放管服效”改革。以企业投资项目承诺制为牵引推进审批服务便民化，全面推开“证照分离”“多证合一”改革工作，完善推广“双随机、一公开”智慧综合监管平台。引深“互联网+政务服务”，重点领域和高频事项基本实现“一网一门一次”，市县两级审批服务事项80%以上可网上办理，打造“六最”营商环境，营商环境评估要走在全省前列。

进一步支持民营经济创新发展。要落实国家、省各项减税降费政策，坚决治理乱收费和乱罚款，让企业轻装上阵。要落实我市支持民营企业发展30条，完善配套举措，确保政策落地见效。要发挥好政银企合作平台作用，创新“助保贷”融资模式，切实解决民营企业融资难问题。要加大“小升规”培育力度，年内规上企业力争突破500家。引导中小企业走“专精特新”发展之路，打造一批民企“小巨人”。要依法保护民营企业、企业家的合法财产不受侵犯、合法经营不受干扰，设立民营企业政策性救助专项资金，帮助重点民营企业纾困，让民营企业家吃下定心丸，安心谋发展。

各位代表，习近平总书记在庆祝改革开放40周年大会上的重要讲话，发出了新时代改革开放再出发的动员令。要进一步提高改革开放的本领和能力，谋划发展新思路，出台改革新举措，开辟改革开放新天地，让人民群众共享更多改革开放成果！

（四）全力建设高品质城市，以崭新形象迎接二青盛会。坚持走以质取胜的路子，坚持“内”“外”互动，创新手段与制度，打造功能完善、绿色智慧、管理科学的高品质城市。

以先进理念完善城市规划。实施城市“双修”战略，围绕建设富有特色的国家区域中心城市，提升太原都市区的吸引力和辐射力，高起点编制新一轮城市发展战略规划。进一步优化城市空间布局，拉大城市框架，推动产城融合，促进生产空间集约高效、生活空间宜居适度、生态空间山清水秀。加强城市设计，着力打造重点片区，建设汾河活力轴、西山文化带，复兴府城历史风貌区，塑造城市新地标。

以精品标准推进城市建设。推进交通基础设施建设，提升太原国家级枢纽城市地位。地铁2号线年内实现“轨通”“电通”，确保2020年底开通运营，加快1号、3号线前期工作，做好太原都市区轨道交通线网规划。要推进太焦铁路建设，尽快实现太原至郑州两小时交通圈，开展太原至绥德铁路项目前期研究。加快西北二环高速公路建设，建成后将现有环城高速作为快速路服务城市交通。6月底前，完成108国道、新店街快速化改造、通达桥、古城桥、迎宾桥等市政续建工程，高铁南站东广场、太原站东广场、汽车客运东南站投入运营，合理设置过街天桥和地下通道，方便市民出行。要加强公共服务设施建设，提高水电气热供应保障能力，加快东峰热源厂等项目建设，新增集中供热能力800万平方米，加快推进长风西街、钢园路供水加压站和呼延水厂二期等建设，推进燃气居民用户设施升级改造，完成太原北500千伏输变电等19项重大电网工程。加强通信设施建设，落实全省通信基础设施三年行动计划，加快5G布局和商用进程，在二青会上进行标志性体验。

以“绣花”功夫做好城市管理。坚持以创建全国文明城市为引领，进一步提升市政设施运行和公共空间、交通、环境、应急管理水平，推动“九乱”专项整治重心由点上示范转向面上治乱，成片区整治小街小巷，彻底清除卫生死角，打扫干净屋子迎宾客，展示美丽太原新形象。要统筹利用好地上与地下空间，推进海绵城市建设，加快晋源东区二期地下综合管廊建设。要推进“厕所革命”，抓好垃圾处理设施建设，解决停车难问题，加快老旧小区节能改造，持续改善居住环境。进一步提升市民文明素质，让“爱省会、建太原、树形象”成为全市人民的自觉行动。

以一流水平精心办好二青会。办好二青会，是省委、省政府交给太原的重大任务，我们要举全市之力、倾全城之情，既简约节约，又出彩出色，把二青会办成一届精彩、惠民、难忘的体育盛会。要加快比赛场馆建设收尾，完善场馆周边路网，做好配套设施安装调试，提前制定突发事件、应急救援、紧急避险等安全预案。要精心组织圣火采集、火炬传递、开闭幕式等大型活动，统筹做好参赛备战、竞赛组织、综合保障、青运村运营管理和志愿者服务等各项工作。要借势办好环太原国际公路自行车赛暨中国太原国际自行车周、太原国际马拉松赛、汾河龙舟公开赛等赛事，打造特色体育名片，积极创建全民运动健身模范市。

各位代表，城市是人民追求美好生活的有力依托，要以“功成不必在我”的精神境界和“功成必定有我”的历史担当，一张蓝图绘到底，一任接着一任干，更高起点、更高目标、更高层次绘好新时代“太原画卷”！

（五）全力实施乡村振兴战略，推动农业农村现代化。聚焦全面小康目标，统筹抓好“五个振兴”，突出城郊型地域特色，促进城乡融合发展。

大力发展城郊型都市现代农业。要全力推动南部城郊农业示范区、北部有机旱作特色农业示范区和西部特色农业示范区发展。要扶持壮大农业龙头企业，扩大清徐老陈醋、九牛鲜奶、晋源花卉、阳曲小米、古交榛子、娄烦马铃薯、晋祠大米等特色品牌市场影响力。加快发展“互联网+现代农业”，培植一批电商产业园示范村镇。要实施乡村旅游精品工程，积极发展农耕体验、农业创意、乡村手工艺等新产业，规范有序发展田园综合体，打响省城休闲农业品牌。要严格落实粮食安全责任制和“菜篮子”市长负责制，确保市场供应和价格稳定。

全面改善农村人居环境。要学习浙江“千村示范、万村整治”经验做法，全力做好村庄风貌管控、拆违治乱、垃圾处理、污水治理和农村“厕所革命”等五项重点工作，文明城市创建向农村延伸，改善农村面貌，打造美丽乡村。积极推进“四好农村路”建设，巩固提升饮水安全。要毫不松懈抓好非洲猪瘟疫情防控工作，防范疫情复发反弹。要深入开展“大棚房”专项整治行动，完善标本兼治长效机制，严防死灰复燃。

不断深化农村各领域改革。继续完善农村承包地“三权分置”改革。稳慎推进农村宅基地改革，适度放活宅基地和农民房屋使用权。扎实推进农村集体产权制度改革，6月前基本完成清产核资任务，年底完成身份确认。鼓励和支持人才、政策、资金等向乡村流动，促进更多工商资本、社会资本投资农业农村。继续深化林权、水权等制度改革。

（六）全力打好三大攻坚战，为决胜全面小康打下坚实基础。打好三大攻坚战，事关经济发展质量和小康社会建设全局，必须全力攻坚，扎实推进，务求实效。

坚决防范化解重大风险。落实省委坚持底线思维着力防范化解重大风险专题研讨班会议精神，主动扛起防范化解重大风险的政治责任，坚持底线思维，增强忧患意识，提高防控能力，守土有责、守土负责、守土尽责。要防范金融市场异常波动，深入推进防范和处置非法集资专项行动，扎实开展互联网金融和各类交易场所专项整治，严格规范小额贷款公司和融资性担保公司经营行为。要防范化解政府债务风险，强化预算约束，严格规范政府举债行为，不碰红线、不越底线，完善统计报告和动态监控预警机制，重大项目开工前必须进行财政承受能力评估论证，坚决遏制隐性债务。

巩固提升脱贫成效。坚持精准扶贫精准脱贫基本方略，焦点不散、靶心不变、力度不减，确保全面打赢脱贫攻坚战。要持续开展“城区包乡、单位包村”对口帮扶，巩固提升阳曲脱贫成效，实现娄烦高质量脱贫摘帽，确保2个贫困村800贫困人口全面脱贫。要研究解决收入水平略高于建档立卡贫困户的群体缺乏政策支持等问题，及早谋划脱贫攻坚目标实现后的政策配套、返贫预警等工作，减少和防止脱贫人口返贫。

持续改善环境质量。树牢绿水青山就是金山银山的理念，坚决扛起生态环境保护政治责任，推动省城环境质量改善取得更大成效。要坚决打赢蓝天保卫战，持续推进治污、控煤、管车、降尘。推进工业企业污染深度治理，坚决关停不符合环保标准的工业窑炉等生产设施，以环保倒逼产业升级，支持太钢率先完成超低排放改造，为全市工业企业减排树立标杆；实施农村清洁供暖改造3万户以上；持续提升铁路货运比例，开展油品质量专项整治；严格落实建筑工地“六个百分百”，抓好城乡结合部道路扬尘整治。要坚决打赢碧水保卫战，细化河（湖）长制六大任务，加快建成区雨污分流、老旧管网改造，汾东污水处理厂上半年投运，环卫产业示范基地污泥处理厂、垃圾焚烧电厂、污水处理厂8月投运，加强入河排污口监管，推动河湖水域环境持续好转。要坚决打赢净土保卫战，落实《土壤污染防治法》，加强农业面源污染防控，推进工业固废治理。要大力推进生态建设，汾河治理三期工程6月全段通航，打造河水清新明亮、景色美丽宜人、两岸绿树成荫的靓丽名片。加快东西北山造林绿化提档升级，积极推进晋阳湖景区、植物园、动物园、摄乐公园等公园绿地建设。要坚决打好秋冬防攻坚战，压实监管责任和属地责任，加强预警预判、精准治污，做好重污染天气应对，确保市区 PM2.5平均浓度同比下降2.5%，坚决完成国家下达的改善任务。要以问题整改销号为最终目标，持续推进中央、省环保督察反馈问题整改。坚持环保倒逼转型不动摇，实现经济发展与环境保护协同共赢，努力让广大市民呼吸上新鲜空气、喝上干净的水，让绿色成为太原的底色！

（七）全力增进民生福祉，不断提升人民群众获得感幸福感安全感。坚持以人民为中心的发展思想，着力保障和改善民生，全力办好老百姓关心的事、身边的事。

实施就业优先政策。贯彻落实省政府《关于做好当前和今后一个时期促进就业工作的实施意见》及相关配套政策，积极争取和统筹使用好就业补助资金，确保新政策全面落地。完善稳就业的政策体系，扎实做好高校毕业生、就业困难人员、退役军人等重点群体就业工作。积极支持创业孵化基地和创业园区发展，鼓励以创业带动就业。开展全民技能提升工程，加强全方位公共就业服务，提高劳动力就业能力，确保零就业家庭动态清零。

办好人民满意教育。教育是民生，更是国计。要统筹推进各类教育协调发展，抓好五项重点工程。学前教育普惠发展工程，针对“公办园少、民办园贵”问题，做好幼儿园配建工作，建成34所普惠性幼儿园，新建小区配套幼儿园要办成公办园或普惠性民办园，学前教育普惠率达到85%。义务教育改革发展工程，统筹配置城乡教育资源，推进城乡义务教育一体化发展，认真落实“局管校聘”改革任务，加快乡镇寄宿制学校建设，办好乡村小规模学校，大力推进办学模式改革，促进公办民办学校协调发展，持续增加优质学位，缩小城乡校际差距，着力化解“大班额”“择校热”难题。普通高中提升发展工程，积极推动高中特色化、多样化办学，加快推进新校建设，五中、成成中学等新校区9月投入使用，外国语学校、第二外国语学校新校区全面竣工。职业教育整合发展工程，结合太原产业发展和高技能人才需求，推进职业教育规模化发展，启动建设职教园区，大力培育实用技能型人才。高等教育提速发展工程，对标先进城市，加快市属高校建设步伐，推进太原学院专业优化调整，城市学院实训楼年内投入使用，提高本科教育水平。同时，持续加强教师队伍建设，不断提高教师待遇，让尊师重教蔚然成风。通过三年努力，加快教育现代化，建设教育强市。

提升全民健康水平。加快医疗卫生基础设施建设，市中心医院、妇幼保健院新院区7月投入运行，人民医院一期10月建成。深化医药卫生体制改革，持续推动县乡医疗机构一体化改革走在全省前列。加大高层次医疗卫生人才引进和专家队伍建设，开展改善医疗服务新三年行动计划，健全预约诊疗、远程诊疗、临床路径管理等制度，切实提升人民群众就医获得感。

提升公共文化服务水平。扎实推进媒体融合向纵深发展，不断提高太原媒体的传播力、引导力、公信力。加快推进丁果仙大剧院改扩建项目。深入推进文化惠民工程，继续做好文化消费试点工作，持续开展免费送戏下乡、农村电影放映、全民阅读等活动，丰富精神文化产品，满足人民美好生活需要。

健全住房保障体系。坚持房子是用来住的、不是用来炒的定位，构建多主体供给、多渠道保障、租购并举的住房制度。继续加大城中村、棚户区改造力度，新开工保障房5913套，加快回迁安置房建设和分配，持续改善市民居住条件。培育租赁市场，规范住房开发企业、中介机构销售行为，简化、优化流程，解决房产证办证难问题。

做好社会保障工作。继续实施全民参保计划，稳步提高各项社会保险待遇水平。研究出台加强城市特殊困难群众救助帮扶的政策措施，提高低保、特困人员供养标准，保障下岗失业、就业困难等群体基本生活。加快老年福利院建设，儿童福利院、康宁医院年内建成投用。持续引深全国居家和社区养老服务改革试点工作，全力打造以居家为基础、社区为依托、机构为补充、医养相结合的养老服务体系。深化双拥共建，加强退役军人的保障和服务工作，落实好接收安置、教育培训、抚恤优待等政策。严格落实治欠保支各项制度，保障劳动者合法权益。

抓好安全稳定工作。全面贯彻总体国家安全观，提高风险预知预警预判能力，紧盯重大敏感节点，做到“零懈怠”“零疏漏”“零失误”。要严格落实各类安全生产责任制，重点落实好省下放8座煤矿属地监管职责，抓好瓦斯和水害防治，建立安全生产长效机制，坚决遏制重特大事故。要深入开展扫黑除恶专项斗争，维护社会和谐稳定。要完善应急管理体制机制，不断增强应急保障和处置能力。要进一步强化市场监管，保障食品药品安全，让老百姓吃得放心、穿得称心、用得舒心。

各位代表，民之所盼，政之所向。要从人民群众关心的事情做起，从让人民满意的事情抓起，面对群众心声和美好期盼，真心回应，倾心投入，在发展中补齐民生短板、促进社会公平正义，努力交出一份群众满意的民生答卷！

三、全面加强政府自身建设

新时代政府工作要有新气象新作为。要把民心作为最大政治，把人民满意作为最高追求，不辱时代使命、不负人民重托，更加注重加强自身建设，提升政府治理能力现代化水平，打造为民务实清廉高效政府。

坚持正确政治方向。旗帜鲜明讲政治，树牢“四个意识”，坚定“四个自信”，坚决做到“两个维护”，自觉在政治立场、政治方向、政治原则、政治道路上同以习近平同志为核心的党中央保持高度一致。强化政府党组政治建设，严格执行新形势下党内政治生活若干准则，增强党内政治生活的政治性、时代性、原则性、战斗性。落实民主集中制，提高科学决策、民主决策水平。

全面加强依法行政。弘扬宪法精神，自觉运用法治思维、法治方式开展工作，努力把政府各项工作纳入法治化轨道。落实国家机关“谁执法谁普法”责任制，完善公共法律服务体系，建立健全行政规范性文件管理制度和合法性审查机制，依法办理行政复议和行政应诉案件。严格执行人大及其常委会的决议决定，认真办理人大代表建议、政协提案，自觉接受人大、政协监督以及社会、舆论监督。全面推进政务公开和政府信息公开，重点领域实现“应公开、尽公开，应上网、尽上网”，确保权力在阳光下运行。

提升政务服务水平。以本次机构改革为契机，优化政府机构设置和职能配置，形成职责明确、依法行政的政府治理体系，增强政府公信力和执行力。把“用户思维、客户体验”理念融入政府治理和服务全链条，更多地从群众和企业角度想问题、办事情、定政策、抓落实，不断提高市民对政府服务的认同度和美誉度。要发挥行政审批管理局“一局两中心”职能作用，实现“一枚印章管审批”，推动审批智能化、服务自助化、办事移动化。进一步整合各类政务服务热线，打造全国一流的12345热线便民服务平台。

持续推进廉政建设。全面落实从严治党主体责任，认真履行“一岗双责”，以自我革命的精神持续推动政府系统党风廉政建设。持之以恒落实中央八项规定精神，时刻防范“四风”问题隐形变异新动向，坚决破除形式主义、官僚主义。坚持用制度管权管事管人，加强党风廉政教育，推进廉政文化建设，坚持严字当头，全面从严、一严到底，让铁规铁纪成为政府工作人员的自觉遵循，以清正清廉为干事创业添锐气、增底气。

各位代表，一分部署，九分落实。要认真开展“不忘初心、牢记使命”主题教育，用党的创新理论武装头脑、指导实践、推动工作，努力打造忠诚干净担当的干部队伍。要扎实开展“改革创新、奋发有为”大讨论，按照省委、市委的部署要求，聚焦“六个破除”“六个着力”“六个坚持”，不断增强改革决不能落后的信念、创新驱动发展的理念和勇于担当作为的自觉，努力达到理念的大提升、本领的大提升、作风的大提升，带动整体工作取得“六个新突破”，在并州大地汇聚起追梦奋斗、振兴崛起的强大正能量。要保持斗争精神、增强斗争本领，不断在重大斗争中增强党性、磨练意志、砥砺品格，始终保持共产党人勇于斗争的风骨、气节、操守、胆魄。要增强抓落实的刚性，大力推行一线工作法，持续深化“13710”工作制度，坚持网络节点管理，构建横向到边、纵向到底的责任体系，紧盯重点领域和关键环节，以“钉钉子”精神，以“踏石留印、抓铁有痕”的韧劲，抓住不放、一抓到底、抓出成效，确保各项工作见根见果、见实见效。

各位代表，“我们都在努力奔跑，我们都是追梦人”。让我们更加紧密地团结在以习近平同志为核心的党中央周围，按照省委、省政府的决策部署，在市委的坚强领导下，迎难而上、锐意进取，只争朝夕、真抓实干，以新时代奋斗者的开拓精神，奋力谱写文明开放富裕美丽太原新篇章，以优异成绩庆祝中华人民共和国成立70周年！
